# Supplementary material for: Dengue severity by serotype and immune status in 19 years of pediatric clinical studies in Nicaragua
Source: PLoS Negl Trop Dis. 2025 Jan 10;19(1):e0012811. doi: 10.1371/journal.pntd.0012811 (PMC11750095; doi:10.1371/journal.pntd.0012811)
Supplement: Attachment — Submitted filename: Response to Reviewers Final.docx [file pntd.0012811.s002.docx]

**Reviewer comments**

There are suggestions from the reviewers that can improve the importance of the manuscript and some corrections or modifications.

1) As the incidence severe dengue shifts to older ages, it is important to comment how this data can be extrapolated to older patients:

*Response:* Our studies are primarily focused on children; one of the reasons for this is that historically, children have been the most affected population in Nicaragua, as in many regions worldwide. If the Reviewer's question pertains to the proportion of secondary cases, which is generally higher in the adult population, our cohort studies have documented an overall seroprevalence of 70% at 10 years of age, progressively increasing to 83% by age 17. Specifically, in this study, 1,725 dengue patients were secondary cases, representing 65.6% of the study population. We hypothesize that these children, who have previously encountered DENV, could exhibit immunological responses similar to those of adults. Nevertheless, we still consider that differences may exist, as dengue epidemiology in Nicaragua suggests that adults may experience more than two DENV infections, which could influence the progression of the disease. Further, adults are more affected by comorbidities than younger populations, and this can also influence dengue disease outcome.

A paragraph on this topic was included in the main text (lines 321-331):

“Another potential limitation is that our studies are primarily focused on children, since historically, children have been the most affected population in Nicaragua, as in many regions worldwide. This may reduce the generalizability of our findings to the adult population. However, our studies have documented an overall DENV seroprevalence of 70% at 10 years of age, progressively increasing to 83% by age 17. Specifically, in this study, 1,725 dengue patients were secondary cases, representing 65.6%. We hypothesize that these children, who have previously encountered DENV, could exhibit immunological responses similar to those of adults. Nevertheless, we still consider that differences may exist, as dengue epidemiology in Nicaragua suggests that adults may experience more than two DENV infections, which could influence the progression of disease. Additionally, adults have more comorbidities that can impact dengue disease severity (e.g., obesity, diabetes, hypertension) [45,46]”

2) To investigate correlation of the incidence with the severe cases with the level of a circulating DENV serotype in a season. If appropriate, apply some adjustment to consider for this potential bias.

*Response:* In response to the Reviewer’s and Editor’s suggestion, we have added an analysis of epidemic season in relation to severity in Supplementary Figure 4B, summarized on lines 190-191:

“Additionally, when adjusting for epidemic season, similar results were obtained (Supplementary Figure 4B).”

3) Furthermore, in our studies over the 19 years, generally one serotype predominates the dengue cases in a season, as shown in Supplementary Figure 1; except for DENV4, which only circulated recently after an absence of 30 years, the other serotypes presented roughly similar proportions, with DENV2 predominating in 37.7% of cases, DENV3 in 28.9%, and DENV1 in 21.2%. Since DENV2 was the predominant serotype over the 19 years of the study but was not the most severe (DENV3 was found to be most associated with severity using both WHO classifications), we also consider that severity is not directly correlated with the number of positive cases of dengue that each serotype contributed.one other point is to expand the discussion about the implications of these findings in different epidemiological settings. In general vaccinologists and government officers have not consider the differences in pathogenicity among serotypes, and sequence of infection in analyzing their vaccine safety and efficacy

*Response:* We agree that these results should be considered in the design and use of new vaccines; it is critical to take into account the epidemiological context of different sites, the different pathogenicity of the serotypes, genotypes and lineages, the sequence of infections, and the immune status of the population potentially to be vaccinated. We have included the following paragraph in the Discussion (lines 290-302):

“Our findings have implications for dengue vaccines. Severe dengue can occur in infections by each of the four serotypes; in our study, the most severity was observed in DENV2 and DENV3 infections. Further, severe disease occurs in secondary infections by all serotypes and in primary DENV1 and DENV3 infections. However, currently registered vaccines provide imbalanced protection; Dengvaxia® did not provide protection against any serotype in seronegative participants and showed most efficacy against DENV4 [7,45]. QDENGA® is a strong DENV2 vaccine but does not protect against DENV3 in seronegative recipients, and there was not enough information in the clinical trials on protection against DENV4 [8]. A vaccine that provides balanced protection continues to be an important public health need. Beyond serotype, it is important to consider genotype, as vaccine efficacy can differ by genotype and lineage [46,47]. The findings from this study are specific to the circulating genotypes in Nicaragua (DENV1 GV; DENV2 Asian-American GIII; DENV3 Indian-subcontinent GIII; DENV4 GII), and clinical presentation may be different with other genotypes. Thus, future research is needed to analyze severity by serotype with other genotypes and lineages.”

**Reviewer 1**

The manuscript has well defined objectives, totally aligned with the methodology to test the hypothesis.

The size of the samples is actually very good and allows to achieve more of the results. Nevertheless, the cohort studied only considered child up to 17 yo; these might represent some bias, and it should be explained if these results may be extended to adults where the impact of dengue transmission might be higher.

*Response:* Please see our response above. We focus on the pediatric population, as historically, children have been the most affected population in Nicaragua, as in many regions worldwide. As adolescents have experienced prior DENV infection(s), they may be more similar to adult populations; however, adults will likely experience multiple prior DENV infections and, importantly, be affected by comorbidities (e.g., obesity, diabetes, etc.). A paragraph on this topic was included in the main text (lines 321-331):

“Another potential limitation is that our studies are primarily focused on children, since historically, children have been the most affected population in Nicaragua, as in many regions worldwide. This may reduce the generalizability of our findings to the adult population. However, our studies have documented an overall DENV seroprevalence of 70% at 10 years of age, progressively increasing to 83% by age 17. Specifically, in this study, 1,725 dengue patients were secondary cases, representing 65.6%. We hypothesize that these children, who have previously encountered DENV, could exhibit immunological responses similar to those of adults. Nevertheless, we still consider that differences may exist, as dengue epidemiology in Nicaragua suggests that adults may experience more than two DENV infections, which could influence the progression of disease. Additionally, adults have more comorbidities that can impact dengue disease severity (e.g., obesity, diabetes, hypertension) [45,46]”

Although the conclusions are coherent and well discussed, I think there are some limitations to be addressed. The cohort is limited to child ranging less than 1 to 17 yo; for different reasons, dengue incidence is usually higher in young-adult populations, who actually might had been infected more than once (or several times), therefore the results may (or may not) be different.

Also, it is not clear how the circulation of the different DENV serotypes were by the time of the sampling; if we have a predominant circulation of DENV3, it is expected that more of the severe cases are for DENV3. This information might be important to enrich the discussion.

The public health relevance is established. One of the most important issues is in regards of the vaccines that differentially protect for different serotypes. Perhaps, having a comment on how to tackle this issue would be interesting.

*Response:* The responses to the three main comments from the Reviewer are outlined at the beginning of this document. Additionally, they have been included in the Discussion to enrich it.

The manuscript and the work is solid; the size of the cohort is impressive, and allows a lot of good quality data to be collected and analyzed.

There are some limitations that I think should be considered and discussed. The cohort is limited to child ranging less than 1 to 17 yo; for different reasons, dengue incidence is usually higher in young-adult populations, who actually might had been infected more than once (or several times), therefore the results may (or may not) be different. Some discussion to explain how these results may or may not be extended to other populations, including possible different proportions on severity might be useful.

On the other hand, it is not clear how the circulation of the different DENV serotypes were by the time of the sampling; if we have a predominant circulation of DENV3, it is expected that more of the severe cases are for DENV3. Perhaps a map or a table indicating the proportion of each serotype circulating through the time might be interest and will facilitate the data analysis.

*Response:* The responses to the Reviewer’s main question was included at the beginning of the document and incorporated into the main text Discussion. Additionally, although Figure 1 illustrates the circulation of different DENV serotypes over time, we have included Supplementary Figure 1 that displays only serotype circulation, to enhance clarity for the reader.Finally, one of the most relevant conclusions are about the vaccines and the unbalanced protection against different serotypes. Nevertheless, the discussion around this issue is vague, and more can be elaborated in terms of how we can expect and impact if the vaccination is massive in different settings (high, medium and low endemicity, for instance) and some ideas on how this issue can be (eventually) addressed.

*Response:* Please see response above. We have included a paragraph in the discussion addressing the relevance of our findings in the context of vaccines Discussion (lines 290-302).

**Reviewer 2**

The manuscript entitled “Dengue severity by serotype and immune status in 19 years of pediatric clinical studies in Nicaragua” by Narvaez et al. investigates the relationship of viral serotype and pre-existing immunity to dengue with dengue disease severity in children aged 6 months-17 years old.

The manuscript explores a very relevant topic in dengue disease and has a detailed description of the data and the analysis used for reaching the conclusions. Although this topic has been previously explored in several studies in the dengue field, the main strengths of this study are the comparison of both WHO classifications of dengue severity, from 1997 and 2009, and the analysis of 2,630 community-based and hospital-based cases over a period of almost two decades.1-Please, clarify the age of participants. In line 32, participant age is 6 months-17 years old; in lines 50 and 97, participant age is 2-17 years old; and in table 1, age group <1 years is included.

*Response:* We apologize for the confusion. The differences are due to the fact that we have included two different studies. In the PDHS study, the age range is from 6 months to 14 years, and in the PDCS study, it is from 2 to 17 years. For this reason, there is no change on line 97, as it is describing the age range of the PDCS. A correction was made on line 50, changing the age range from 6 months to 17 years, as this sentence refers to both studies. In Tables 1 and 2, the age group was changed from <1 to 6 to 11 months. To streamline Table 1, columns 1 and 3 were merged as they are part of the same study – column 3 referred to PDCS participants who were transferred to the study hospital. Now column 1 includes all PDCS participants (those who were and were not transferred to the study hospital).

2-In lines 173 to 175, the prevalence analysis of primary and secondary infections according to age group (Table 2) does not include the statistical significance. I suggest including the p-values.

*Response:* As suggested by the Reviewer, the p-values have been included in Table 2.

3-In lines 182-186, the prevalence analysis of primary and secondary infections according to disease severity caused by each serotype also does not include the statistical significance (Table 3 and Figure 2). Likewise, I suggest including the p-values.

*Response:* We would like to clarify that the intention of Table 3 is descriptive in nature to showcase the data. We would prefer not to add the p-values between serotypes, immune status and severity into the table since it would be unwieldly and add too much complexity to the table. However, we have conducted the analysis of comparison between immune status by serotype and severity (using data from Figure 2) and have added the statistical results to lines 187-190:

“The breakdown of severe disease by immune status of DENV1 and DENV3 was significantly different from that of DENV2 (DENV2-DENV1: OR 8.21, 3.58-18.87; DENV2-DENV3: OR 9.41, 4.98-17.78).”

4- Why are two studies included in Supplementary Figs. 2 and 3 (Pediatric Dengue Cohort Study and Pediatric Dengue Hospital-based Study) instead of three studies, as indicated in Table 1 and Supplementary Figs 4-9? Please clarify.

*Response:* We thank the Reviewer for pointing this out. The fact is that we only have 2 studies (the PDCS and the PDHS), the confusion is that third “study” consisted of only the participants in the PDCS who were transferred from the study health center to the study hospital (the PDHS). To clarify and streamline, we have removed the separate column of transferred participants and retained the information as PDCS (those who were and were not transferred to the study hospital), PDHS, and the combination of both studies.

5-Did the comparative analysis of community-based and hospital-based cases yield any difference in clinical signs and symptoms of dengue and laboratory results?

*Response:* Although the comparison between the PDCS and PDHS cohorts was not an objective of our study, we can provide some data in response to the Reviewer’s question. According to the 1997 classification, 25% of PDHS cases exhibited severity, compared to only 6% in the PDCS (p<0.0001), as the latter is community-based and thus has a preponderance of milder cases. Regarding the 2009 classification, 21% of PDHS cases exhibited severity, compared to 12% in the PDCS (p<0.0001). Regarding individual signs, such as thrombocytopenia, it was observed in 43% of PDHS cases and 21% of PDCS cases, with 15% and 4%, respectively, showing platelet count below 50,000. When analyzing by serotype, those with lesser severity, such as DENV1 (22% in PDCS vs. 20% in PDHS) and DENV4 (17% in PDCS vs. 7% in PDHS), were reported to a greater extent in the PDCS. In contrast, DENV2 (40% in PDHS vs. 36% in PDCS) and DENV3 (34% in PDHS vs. 24% in PDCS) were found to a greater degree in the PDHS.

6-Figures 4 and 5 and Supplementary Figs 4-9. The number of events and observations from Supplementary Figures 4-9 (stratified by immune status and study) do not match those from Figures 4 and 5. Please clarify. For example, in Fig 4 there are 33 events of hypotensive shock in 557 DENV-1 infections, while in Supplementary Figs 4-9 there are 66 events of hypotensive shock in 1114 DENV-1 infections (considering primary and secondary infections and the three studies). The same happens with the other clinical variables.

*Response:* We apologize, we had erroneously labelled supplementary figure 5 and 8 as participants transferred from the PDCS to the PDHS; in fact, these represented the combination of the PDCS and PDHS studies. We have corrected the figure legend, and now the numbers should make sense. Again, we apologies for this confusion. Figures 4 and 5 (PDCS+PDHS irrespective of immune status) now match Supplementary figures 5 and 8 (PDCS+PDHS, stratified by primary and secondary infection status).

7-In lines 207-213, the analysis of compensated and hypotensive shock by DENV serotype according to data from Table 4 should include the statistical significance of the results, indicating the p-values.

*Response:* The statistical significance and p-values have now been included in the main text and in Table 4.

8-Table 1, include “Age (years)” and “Sex” (instead of sexo) in the first

*Response:* We have made the corrections suggested by the Reviewer.

9-Fig. 1, the maximum value on the y-axis scale should be included (600).

*Response:* The maximum value for the y-axis scale has been included, as suggested.

10-Table 3, correct “DWW” to “DWWS” in the first column.

*Response:* We have made the correction suggested by the Reviewer.

11-Lines 48-59, Summary section states the study of natural infections in pediatric patients. Have any of the participants been vaccinated with any dengue vaccines prior to study? Please, clarify.

*Response:* None of the study participants have been vaccinated with the currently available dengue vaccines.

12-Lines 121-123 – Methods. Why were multiple different PCR methods used instead of just one consistently? Lanciotti initially used, then real time flavi-multiplex followed by real time denv serotype panel.

*Response:* Between 2004 and 2009, the RT-PCR methods described by Lanciotti and Harris were used. After the H1N1 pandemic, when real-time PCR was expanded throughout the region, we began to use this technology in Nicaragua. Thus, initially, we used the RT-PCR assay described by Lanciotti, and later, up to the present, we have used the multiplex real-time RT-PCR designed by Dr. Jesse Waggoner to detect dengue, chikungunya, and Zika viruses (refs 28, 29, 30, and 31). Since this publication reports on 19 years of our studies, the molecular techniques used naturally progressed along with new developments in molecular diagnostics.

13-Lines 268-280, 282-287 - Paragraphs review the use of WHO definitions and indicate the need for improved WHO disease severity definitions. Should have one, all encompassing system to clearly define disease, not two different classifications.

*Response:* While our primary objective was not to compare or improve these classifications — given that many articles have already addressed this — we recognize the value of both. Since the results differ, we chose to base our analysis on both classifications. The 1997 classification focuses on the pathophysiology of dengue, while the 2009 classification is more clinically oriented.

14-Line 478 - Table 1 is missing “Sex: Male” demographic information?

Lines 505-507, 526 - Figure 1 Legend needs to define “S” versus “P” serotypes.

*Response:* We have made the corrections suggested by the Reviewer.

15-Lines 509-510, 528 – Figure 2 Legend again needs to define “S” versus “P” and “IR” … I assume IR is Immune Response. Please, further clarify for readers. Suggest simplifying x-axis serotype names for improved reading, eg, “DENV1” to DV1” to help with spacing.

*Response:* We have made the corrections suggested by the Reviewer. However, we chose to retain the “DENV” designation, as this is the officially accepted designation.

16-Lines 512-516, 531 – Figure 3 legend clearly defines abbreviations “IR” and “S” and “P”. Please clarify these abbreviations on Figures 1 and 2 as well. Why is DENV1 automatically named the reference value?

*Response:* As mentioned above, we have now defined IR, S, and P in the legends to Figures 1 and 2, as appropriate. DENV4 is often considered the least severe serotype followed by DENV1. We did not use DENV4 as the reference due to low case counts in our study because of its extended absence and recent re-introduction. As such, we used DENV1 as the reference. Using the least severe serotype as the baseline provides a natural comparator for studying risk or severity gradients across serotypes. Additionally, prior cross-reactive immunity protects against DENV1 (Zambrana et al. 2024, Katzelnick et al. 2020), which makes DENV1 a logical reference for evaluating serotype-specific risks among secondary infections.

Zambrana JV, Hasund CM, Aogo RA, Bos S, Arguello S, Gonzalez K, Collado D, Miranda T, Kuan G, Gordon A, Balmaseda A, Katzelnick LC, Harris E. Primary exposure to Zika virus is linked with increased risk of symptomatic dengue virus infection with serotypes 2, 3, and 4, but not 1. Sci Transl Med. 2024 May 29;16(749):eadn2199. doi: 10.1126/scitranslmed.adn2199. Epub 2024 May 29. PMID: 38809964.

Katzelnick LC, Narvaez C, Arguello S, Lopez Mercado B, Collado D, Ampie O, Elizondo D, Miranda T, Bustos Carillo F, Mercado JC, Latta K, Schiller A, Segovia-Chumbez B, Ojeda S, Sanchez N, Plazaola M, Coloma J, Halloran ME, Premkumar L, Gordon A, Narvaez F, de Silva AM, Kuan G, Balmaseda A, Harris E. Zika virus infection enhances future risk of severe dengue disease. Science. 2020 Aug 28;369(6507):1123-1128. doi: 10.1126/science.abb6143. PMID: 32855339; PMCID: PMC8274975.

17-Lines 518—521, 534 – Figure 4, again, comparing values of different dengue disease manifestations with DENV1 as the reference serotype. Please, clarify further as to why?

*Response:* Please see the response above.

18-Lines 523-524, 538 – Figure 5 also uses DENV1 as the reference serotype. Why?

*Response:* Please see the response above.
